# Experiences of private sector quality care amongst mothers, newborns, and children in low- and middle-income countries: a systematic review

**DOI:** 10.1186/s12913-021-06905-3

**Published:** 2021-12-06

**Authors:** Joe Strong, Samantha R. Lattof, Blerta Maliqi, Nuhu Yaqub

**Affiliations:** 1 Department of International Development, London School of Economics and Political Science, Houghton St, London, WC2A 2AE UK; 2 Department of Maternal, Newborn, Child and Adolescent Health and Ageing, World Health Organization, Avenue Appiah 20, CH-1211 Geneva 27, Switzerland; 3 Child and Adolescent Health Unit, WHO Regional Office for Africa, Cite du Djoue, P.O.Box 06, Brazzaville, Congo

**Keywords:** Systematic review, Private health sector, Maternal health, Newborn health, Child health, Experience of care, Quality of care, India, Bangladesh, Uganda

## Abstract

**Background:**

Experience of care is a pillar of quality care; positive experiences are essential during health care encounters and integral to quality health service delivery. Yet, we lack synthesised knowledge of how private sector delivery of quality care affects experiences of care amongst mothers, newborns, and children. To fill this gap, we conducted a systematic review that examined quantitative, qualitative, and mixed-methods studies on the provision of maternal, newborn, and child health (MNCH) care by private providers in low- and middle-income countries (LMICs). This manuscript focuses on experience of care, including respectful care, and satisfaction with care.

**Methods:**

Our protocol followed the Preferred Reporting Items for Systematic Reviews and Meta-Analyses. Searches were conducted in eight electronic databases (Cumulative Index to Nursing and Allied Health, EconLit, Excerpta Medica Database, International Bibliography of the Social Sciences, Popline, PubMed, ScienceDirect, and Web of Science) and two websites and supplemented with hand-searches and expert recommendations. For inclusion, studies examining private sector delivery of quality care amongst mothers, newborns, and children in LMICs must have examined maternal, newborn, and/or child morbidity or mortality; quality of care; experience of care; and/or service utilisation. Data were extracted for descriptive statistics and thematic analysis.

**Results:**

Of the 139 studies included, 45 studies reported data on experience of care. Most studies reporting experience of care were conducted in India, Bangladesh, and Uganda. Experiences of private care amongst mothers, newborns, and children aligned with four components of quality of care: patient-centeredness, timeliness, effectiveness, and equity. Interpersonal relationships with health care workers were essential to experience of care, in particular staff friendliness, positive attitudes, and time spent with health care providers. Experience of care can be a stronger determining factor in MNCH-related decision-making than the quality of services provided.

**Conclusion:**

Positive experiences of care in private facilities can be linked more broadly to privileges of private care that allow for shorter waiting times and more provider time spent with mothers, newborns, and children. Little is known about experiences of private sector care amongst children.

**Trial registration:**

This systematic review was registered with the PROSPERO international prospective register of systematic reviews (registration number CRD42019143383).

**Supplementary Information:**

The online version contains supplementary material available at 10.1186/s12913-021-06905-3.

## Background

Increasing access to and provision of care without an explicit focus on quality of care limited improvements in maternal, child, and newborn health (MNCH) care during the course of the Millennium Development Goals (2000–2015) [1]. Research over the last decade has revealed that quality of care is an essential element of health care [2] and is necessary to achieve progress towards the Sustainable Development Goals [3]. Evidence from interventions to encourage facility-based births in India [4, 5], Rwanda [6], and Malawi [7] emphasised that quality care, referral systems, supplies, and clinical skills are critical to reducing mortality outcomes. The focus on improving indicators of facility-based MNCH care must be coupled with improvements in the quality of care.

Comprised of six intersecting components, quality of care is care that is safe, effective, patient-centred, timely, efficient, and equitable [8, 9]. Positive experience of care is intrinsic to improved quality of care, and whilst it can be located within patient-centeredness, it intersects multiple components of quality [10, 11]. Improved provision of care impacts a person’s experience of care, whereas positive health seeking behaviours and future decision making are impacted by the experience of care [12]. In maternal health care, evidence links positive pregnancy experiences to higher quality interpersonal exchanges, greater fairness, and greater health worker contact [13]. Respectful maternal care is also a major component of a person’s experience of care [14] and a critical component of quality of care [1].

Experience of care is rarely included as a measure in large-scale facility-assessment tools [1], in part because it has been historically difficult to define and measure [12]. The underlying constructs that constitute experience of care are frequently blurred and inconsistent across literature. Moreover, experience of care is linked to but conceptually separate from satisfaction, and the relationship between these concepts is frequently complicated [15]. Experience of care is a subjective process indicator of quality, while satisfaction is an outcome indicator relative to a person’s expectations of the care they received [8, 9, 14]. Measures such as communication, timeliness of care, choice, and respect impact both experience of care and satisfaction; however, experience of care and satisfaction are only partially associated with each other [16]. Evidence illustrates that measuring both experience of care and satisfaction allows for a greater understanding of the quality of services and provides key information for improving service quality [14].

With regards to MNCH, the World Health Organization (WHO) recognises the complex relationship between experience of care and health outcomes [17, 18]. Experiences of care can impact health outcomes by encouraging people to seek care at particular facilities or seek follow-up care; experiences of care can also be influenced by health outcomes, whereby negative outcomes lead to negative perceived experiences [15, 17, 18]. Moreover, experience of care and satisfaction with care are influenced by factors beyond specific health outcomes, such as the provision of seats in the waiting area, facility hygiene, and expectations of care [19]. Understanding how these various components of experience of care relate to MNCH and can improve respectful, person-centred care is critical for improving quality of care.

As a pillar of quality of care, experience of care appears in the WHO framework for quality of maternal and newborn health care, where it is disaggregated into three critical components: effective communication, respect and dignity, and emotional support [17]. The framework for improving the quality of paediatric care includes similar domains under experience of care: effective communication and meaningful participation; respect, protection and fulfilment of child rights; and emotional and psychological support [18]. An additional component – user-centred health systems – has also been used to understand experience of care [8].

The private sector, which includes individuals and organizations that are neither owned nor directly controlled by governments and are involved in the provision of health services (i.e., for-profit and not-for-profit entities; providers in the formal and informal sectors; and domestic and international actors, charities, faith-based organizations, and non-governmental groups) [20], plays a growing role in delivering MNCH services as well as sexual and reproductive health services [21, 22]. An estimated one in five births in low- and middle-income countries is delivered via the private sector [21]. Yet, the quality of services provided varies [23, 24]. There is a need to address inconsistent quality of care in the private sector and experiences of care more specifically [14]. Despite the importance of experience of care on health outcomes and the links between quality of care and experience of care, there has been no synthesis of experiences of quality MNCH services in the private health sector.

The Network for Improving Quality of Care for Maternal, Newborn and Child Health (the Network), a partnership of 11 countries (Bangladesh, Côte d’Ivoire, Ethiopia, Ghana, India, Kenya, Malawi, Nigeria, Sierra Leone, the United Republic of Tanzania, and Uganda) and their technical partners, was launched in 2017 with the aim of halving maternal and newborn deaths and stillbirths in participating health facilities in 5 years’ time [25]. Network members realize that the private sector has an important role in providing quality MNCH services within mixed (i.e., public and private) health systems. Since 2019, the WHO-based Network Secretariat has been conducting research that aims to fill gaps around how to effectively engage and sustain private sector involvement in delivering quality MNCH care in low- and middle-income countries. As part of this effort, the Network Secretariat conducted a systematic review that addresses four primary research questions:

1. How does the provision of quality health care by the private sector affect morbidity and mortality among mothers, newborns, and children?
2. How does provision of quality health care by the private sector affect utilization of services by mothers, newborns, and children?
3. How effective and efficient is the private sector at delivering quality of care?
4. Among mothers, newborns, and children utilizing health care provided by the private sector, what are their experiences of care? [26]

This study is part of that larger systematic review. Given the extensive amount of data and studies in the entire systematic review, our aim in this article is to answer the fourth research question by systematically assessing the evidence from studies reporting outcome data on experiences of private sector quality MNCH care. Results from complementary analyses on the first three research questions will be presented in separate companion articles.

## Methods

We conducted a systematic review following guidance in the Preferred Reporting Items for Systematic reviews and Meta-Analyses (PRISMA) Statement for clear and transparent reporting of systematic reviews and meta-analyses [27, 28]. As noted in the PICOTS in Table 1, studies reporting on qualitative, quantitative, and/or mixed-methods data from low- and middle-income countries were considered. For inclusion in the systematic review, studies must have examined at least one of the following outcomes: maternal morbidity, maternal mortality, newborn morbidity, newborn mortality, child morbidity, child mortality, service utilization, components of quality of care (i.e. safety, effectiveness, timeliness, efficiency, equity, people-centred care), and/or experience of care, including respectful care. In recognition of the rapid increase in public-private collaborations for health during the late 1990s [29], studies must have been published between 1 January 1995 and 30 June 2019 in English, French, German, or Italian. Ethical approval was not required.

**Table 1:** PICOTS criteria used in the systematic review

PICOTS	
Populations	Pregnant people, mothers, newborns, and children (aged 9 years and under)
Interventions	Delivery of quality maternal, newborn, and/or child health services by the private sector
Control	Not necessary
Outcomes	Quantitative, qualitative, or mixed-methods data on: • maternal morbidity • maternal mortality • newborn morbidity • newborn mortality • child morbidity • child mortality • components of quality care (i.e. safety, effectiveness, timeliness, efficiency, equity, people-centred care) • experience of care, including respectful care • service utilization
Timeframe	1 January 1995 to 30 June 2019
Setting	Low- and middle-income countries

We searched journals from eight electronic databases (Cumulative Index to Nursing and Allied Health, EconLit, Excerpta Medica Database, International Bibliography of the Social Sciences, Popline, PubMed, ScienceDirect, and Web of Science) and two websites (Health Care Provider Performance Review and the Maternal healthcare markets Evaluation Team at the London School of Hygiene & Tropical Medicine). We supplemented these searches with hand searching of reference lists and expert-recommended articles. The searches, application of inclusion/exclusion criteria, screening, and data extraction were conducted using a published protocol and data extraction tools [26]. The search was registered with the PROSPERO international prospective register of systematic reviews (registration number CRD42019143383). Search terms appear in Table 2, and the full electronic search strategy for each database appears in the protocol [26]. Searches were completed on 23 June 2020.

**Table 2:** Search terms and their combinations

1. Private sector	2. Quality of care	3. MNCH
private sector	quality	matern*
for-profit		pregnan*
for profit		mother*
public-private		newborn*
private enterprise*		infant*
NGO		child*
non-government*		pediatric*
		paediatric*
		neonat*

*Refers to truncated word roots in order to capture multiple derivations, e.g. neonat* will capture neonate, neonates, neonatal

Quantitative and qualitative data were extracted on the following categories:

- Background information (e.g., author, date, setting, study objective)
- Intervention background information (e.g., implementing agency, geographic level, study population)
- Intervention details (e.g., intervention recipients, nature of intervention, dimensions of quality care)
- Critical outcomes (both quantitative and qualitative):
  - ◦ Maternal morbidity
  - ◦ Maternal mortality
  - ◦ Newborn morbidity
  - ◦ Newborn mortality
  - ◦ Child morbidity
  - ◦ Child mortality
  - ◦ Service utilization
  - ◦ Experience of care, including respectful care
  - ◦ Components of quality care (i.e. safety, effectiveness, timeliness, efficiency, equity, people-centred care)
- Evaluation/study details (e.g., study type, data type, intervention claims, strategy effectiveness, cost data)
- Study quality (qualitative and quantitative)

JS and SRL extracted and quality assessed studies in duplicate. Quantitative studies were assessed using the Effective Public Health Practice Project quality assessment tool [30], and qualitative studies were assessed using Miltenburg et al.’s quality assessment tool based on criteria developed by Walsh and Downe [31, 32]. The analysis synthesizes data from studies related to experience of care, including respectful care.

Qualitative data were thematically analysed using a three-stage approach, appropriate for systematic reviews [33]. All data were coded with descriptive codes that were in turn collated into broader descriptive themes. Analytic themes were deduced from the returned literature, using measures of experience of care taken from literature as guidance. Measures related to experience of care addressed patient-provider relationships (e.g., patient involvement in decisions about their care and information about their care, feeling isolated, receiving information, provider attention, friendliness of care, confidence and trust in the services received, treatment by doctors, abuse, confidentiality, privacy, communication), client assessments (e.g., client satisfaction, overall satisfaction, rating of consultations, reliability of services, client complaint scores), quality (e.g., interpersonal aspects of quality, perceptions of quality, client quality scores), time (e.g., waiting times, time spent with health care providers, timeliness of care, delays in receiving services), patient experiences (e.g., general experience, care experience, women’s experience of human and physical resources, client preference, seeking alternative care), costs (e.g., costs of care, financial burdens), and facility experiences (e.g., facility cleanliness, seats available in the waiting area, privacy, availability of services, reasons for choosing the facility, returning clients). Within the included studies on experience of care, data on satisfaction with care are included in the results to acknowledge the relational nature of satisfaction as an outcome measure of experience [9].

Given the heterogeneity between the studies in terms of study designs and interventions, it was not possible to conduct a meta-analysis for the outcome experience of care. Quantitative findings are presented using a narrative synthesis with tables of descriptive statistics. More detailed summary tables, including quality scores, appear in Additional File 1. The following findings present descriptive statistics followed by analytic themes.

## Results

### Descriptive statistics

**Fig. 1:**
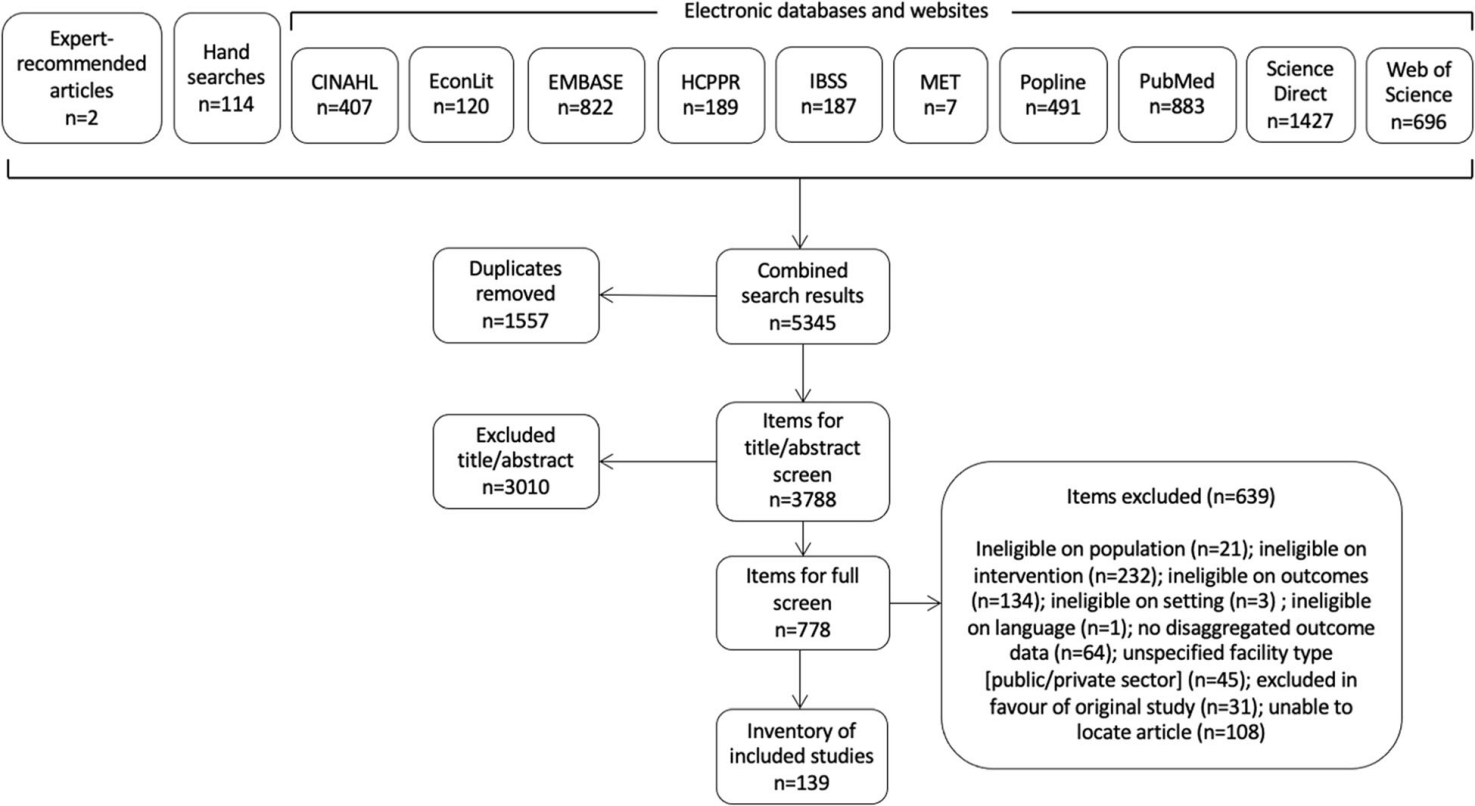
Screening results

As shown in Fig. 1, the search generated 5345 items for screening. After duplicate removal, the 3788 remaining items were screened for inclusion on the basis of title and abstract. Where exclusion could not be determined on the basis of title and abstract, SRL screened the full text. Decisions were made in favour of an inclusive approach if questions remained. Of the 778 full texts screened, 139 studies met all the inclusion criteria and were included in the systematic review.

Studies most frequently reported outcome data on quality of care (*n* = 110) followed by experience of care (*n* = 45) (Table 3). The total number of data points in Table 3 exceeds the number of studies in the final inventory, since some studies presented multiple relevant outcomes. The remaining findings in this article focus on the 45 studies that reported outcome data on experience of care.

**Table 3:** Outcomes of included studies

*Reported study outcomes*	*Number of studies in final inventory that report the outcome*	*Number of studies reporting outcome data on experience of care and the additional outcome(s)*
Maternal morbidity	15	6
Maternal mortality	7	–
Infant morbidity	6	–
Infant mortality	16	5
Child morbidity	14	6
Child mortality	9	3
Quality of care	110	34
Experience of care	45	–
Service utilization	7	2
Infant/child growth^a^	9	2

^a^Secondary outcome

Most studies reporting outcome data on experience of care were conducted in India (24.4%), Bangladesh (11.1%), Uganda (11.1%), and Kenya (6.7%) (Table 4). The majority of studies presented quantitative data (60.0%), with 13.3% of studies presenting exclusively qualitative data and 26.7% of studies presenting both quantitative and qualitative data (Table 5). Over half of studies (51.1%) occurred in countries classified as lower-middle-income. The level of geographic coverage varied with over half of studies conducted at the sub-national level (57.8%) and one-fifth of studies conducted in health facilities (20.0%). Studies exploring experiences of care were largely limited to women seeking care during pregnancy, delivery, and postpartum; only one study asked children about their experiences of care.

**Table 4:** Included studies by region and country

*Region/country*	*Number of studies included in final inventory (%)*	*Number of studies examining experience of care (%)*	*Region/country*	*Number of studies included in final inventory (%)*	*Number of studies examining experience of care (%)*
**Africa**	**49 (35.3%)**	**14 (31.1%)**	**Asia**	**67 (48.2%)**	**24 (53.3%)**
Angola	1 (0.7%)	–	Afghanistan	2 (1.4%)	–
Côte D’Ivoire	1 (0.7%)	–	Bangladesh	11 (7.9%)	5 (11.1%)
Ghana	1 (0.7%)	–			
Ethiopia	2 (1.4%)	–	China	2 (1.4%)	–
Kenya	11 (7.9%)	3 (6.7%)	Georgia	1 (0.7%)	–
Lesotho	1 (0.7%)	–	India	30 (21.6%)	11 (24.4%)
Malawi	3 (2.2%)	–	Indonesia	2 (1.4%)	–
Niger	1 (0.7%)	–	Iran	2 (1.4%)	1 (2.2%)
Nigeria	3 (2.2%)	1 (2.2%)	Jordan	1 (0.7%)	1 (2.2%)
Tanzania	3 (2.2%)	2 (4.4%)	Nepal	4 (2.9%)	2 (4.4%)
The Gambia	1 (0.7%)	1 (2.2%)	Pakistan	6 (4.3%)	2 (4.4%)
Uganda	15 (10.8%)	5 (11.1%)	Philippines	2 (1.4%)	–
Zambia	2 (1.4%)	–	Sri Lanka	2 (1.4%)	–
Multiple countries	4 (2.9%)	2 (4.4%)	Turkey	2 (1.4%)	2 (4.4%)
**Latin America & Caribbean**	**14 (10.1%)**	**6 (13.3%)**	**Oceania**	**1 (0.7%)**	**–**
Brazil	5 (3.6%)	2 (4.4%)	Papua New Guinea	1 (0.7%)	–
Guatemala	2 (1.4%)	2 (4.4%)			
Haiti	2 (1.4%)	1 (2.2%)	**Cross-Regional Studies**	**8 (5.8%)**	**1 (2.2%)**
Mexico	4 (2.9%)	1 (2.2%)			
Multiple countries	1 (0.7%)	–	**Total**	**139 (100%)**	**45 (100%)**

**Table 5:** Characteristics of included studies

*Characteristics*	*Number of studies included in final inventory (%)*	*Number of studies examining experience of care (%)*
**Methodology**		
Randomized controlled trial	1 (0.7%)	–
Controlled clinical trial	1 (0.7%)	1 (2.2%)
Cohort analytic	10 (7.2%)	4 (8.9%)
Case-control	2 (1.4%)	1 (2.2%)
Controlled (before & after)	7 (5.0%)	2 (4.4%)
Interrupted time series	1 (0.7%)	–
Qualitative	8 (5.8%)	6 (13.3%)
Mixed methods	21 (15.1%)	8 (17.8%)
Regression	55 (39.6%)	15 (33.3%)
Other	31 (22.3%)	8 (17.8%)
Unclear / not specified	2 (1.4%)	–
**Country Income Group**		
Low	33 (23.7%)	11 (24.4%)
Lower-middle	75 (54.0%)	23 (51.1%)
Upper-middle	19 (13.7%)	9 (20.0%)
Multiple	12 (8.6%)	2 (4.4%)
**Geographical Level**		
National	34 (24.5%)	5 (11.1%)
Sub-national (e.g. state, city)	73 (52.5%)	26 (57.8%)
Local (e.g. village)	7 (5.0%)	4 (8.9%)
Health facility	18 (12.9%)	9 (20.0%)
Other	5 (3.6%)	1 (2.2%)
Unclear / not specified	2 (1.4%)	–
**Study Population**		
Pregnant women	11 (7.9%)	5 (11.1%)
Women during childbirth	2 (1.4%)	–
Mothers postpartum	12 (8.6%)	3 (6.7%)
Infants	13 (9.4%)	6 (13.3%)
Children	9 (6.5%)	1 (2.2%)
Health care providers	41 (29.5%)	10 (22.2%)
Parents / child caretakers	4 (2.9%)	2 (4.4%)
Multiple answers from list	26 (18.7%)	8 (17.8%)
Other (e.g., urban poor, married women)	20 (14.4%)	10 (22.2%)
Unclear/unspecified	1 (0.7%)	–
**Publication Type**		
Peer-reviewed journal article	103 (74.1%)	35 (77.8%)
Report	27 (19.4%)	8 (17.8%)
Book or book chapter	1 (0.7%)	1 (2.2%)
Other (e.g., conference paper, abstract)	8 (5.8%)	1 (2.2%)
**Implemented a specific intervention beyond the delivery of quality care?**		
Yes	58 (41.7%)	15 (33.3%)
No	81 (58.3%)	30 (66.7%)
**Type of Data**		
Quantitative	104 (74.8%)	27 (60.0%)
Qualitative	8 (5.8%)	6 (13.3%)
Both	27 (19.4%)	12 (26.7%)
**Longitudinal data?**		
Yes	45 (32.4%)	13 (28.9%)
No	90 (64.7%)	30 (66.7%)
Unclear / not specified	4 (2.9%)	2 (4.4%)

Amongst studies reporting data on experience of care, one-third of studies (*n* = 15) implemented a specific intervention (Additional File 2) that went beyond the broad delivery of quality care in the private sector (*n* = 30). These interventions were most often single interventions and focused on supply-side factors (Additional File 2). On-site support for quality improvement was most common intervention (66.7%). Almost all interventions (14/15) directly targeted private health care providers, and they occasionally targeted women during pregnancy, delivery, and postpartum. Interventions to deliver quality rarely targeted infants or children either directly or indirectly. Half of studies implementing specific interventions reported positive claims about the interventions (50.0%) with the remaining studies reporting mixed claims (28.6%) or negative claims (7.1%). In two studies, the authors did not specify the intervention’s success. Only four studies reporting specific interventions were assessed as being strong quality (*n* = 1) [34] or moderate quality (*n* = 3) [35, 36]. Additional details of specific intervention studies appear in Additional File 1 and in the thematic analyses below.

### Mothers’, newborns’, and children’s experiences of and satisfaction with care in the private sector

Among studies reporting experience of quality MNCH care in the private sector, four key themes emerged: (1) comparative studies between public and private health facilities illustrate the relative importance of interactions with health care workers and timeliness in overall experience, and they provide opportunities for shared learning between facilities; (2) mothers’, newborns’, and children’s interactions with health care workers and staff in private clinics most frequently impacted their reported experience of care; (3) timeliness was an essential component of care and associated with both experiences of care and satisfaction with care; and (4) few studies presented findings on contextual inequalities and experiences of private health care beyond affordability. Studies reporting on experiences of care amongst children and newborns did so from the perspective of parents, caregivers, or through clinical observations, rather than from the newborns or children themselves. Thus, the results for newborns and children are experiences of care by proxy.

#### Comparative studies between public and private facilities

Comparative studies of the private and public sectors highlighted that experience of care was not uniformly better in private or public facilities. Rather, both private and public facilities could provide important lessons for quality improvement. In the private sector, women valued their experiences of care for being efficient, timely, clean, and patient-centred. In particular, women obtaining care in the private sector experienced reduced wait times. Women at private antenatal clinics in Pakistan were able to spend an average of 8 min with their health care provider, whereas women at public facilities spent an average of 3 min with their health care provider [37]. For pregnant people attending abortion services in Nepal, experience of counselling was better in private facilities than public facilities, in part due to increased privacy in private settings [38]. Women using maternal health services in urban India had favourable experiences in private facilities, finding private facilities to be cleaner and less crowded than public facilities [39]. Staff treatment was especially important when women required health care with options. Women in Tamil Nadu, India, preferred private clinics for abortion services in order to avoid sterilisation being a condition of their care [40]. Abortion care seekers in Istanbul, Turkey had positive experiences in private facilities, finding the services straightforward and efficient compared to women using public facilities [41].

Results assessing the provision of care in private facilities showed mixed results. While private hospitals delivering maternal and child health services in Nairobi, Kenya provided good staff interactions, public facilities had more experienced staff and higher quality services [35]. Similarly, in a study of primary care experiences in Brazil, better quality interactions with staff in private facilities offset higher quality services at public facilities [42]. Interestingly, despite antenatal care quality being worse at private facilities in Kenya, complaints about staff were higher in public facilities [43]. In a controlled clinical trial of MNCH services in Pakistan, non-governmental organisation (NGO)-contracted rural health facilities had higher client satisfaction scores than government-run rural health facilities; clients at NGO-contracted rural health facilities also reported a higher inclination to deliver within a health facility [44]. These studies indicated that a person’s experience can impact their perception of the quality of care they received, even if the quality of care received was lower than might have been obtained elsewhere, and might impact subsequent health outcomes. They highlighted that the provision of high-quality care alone might not be compelling enough for people who have experienced negative interactions with health care workers or who perceive that they will experience negative interactions with health care workers. Comparative studies indicated that interactions with health staff and timeliness were particularly important factors in mothers’ and children’s experiences of care; these two factors shaped mothers’ and children’s intentions for future care.

#### Interpersonal interactions with health care workers and staff

Studies frequently reported interpersonal communications and interactions that intersected all four domains within experience of care: support, effective communication, respect and dignity, and user-centred health systems. These interactions most often involved the behaviours of health care workers towards mothers, newborns, and children receiving care, including experiences of care during treatment (pre-care, during, and post-care), staff attitudes (real or perceived), and experiences of counselling [38, 45–48]. Interpersonal relationships are an essential component of experience of care, as indicated by evidence from intervention evaluations and reports from care seekers. A microfinance intervention with midwives in Uganda that aimed at assessing the impact of business loans on quality of services included “good handling” as a core measurement of success. While the intervention had no net effect on client “handling,” attending women who reported good experiences of “handling” were 1.8 times more likely to report repeat visits to that facility [49].

Qualitative and observational studies corroborated the significance of interpersonal communications between health care workers and care seekers. Among women accessing reproductive and child health services from franchises in Ghana, positive experiences of care were linked to staff being “sociable” and to staff giving clients space for questions and answers in case of confusion [50]. An observational study of women seeking antenatal care in public and private hospitals in Turkey also reported that the all-women maternity staff at the private hospital had better interpersonal skills with regards to respect and courtesy for care seekers [51]. In Guatemala, a comparison of the provision of care amongst government and private NGOs used “friendliness” as a measure of satisfaction with care; women aged 15–44 and their children reported higher scores of friendliness among NGO services [46].

Women’s ability to communicate with health care staff during consultation, treatment, and provision of care was critical in determining positive or negative experiences. A mixed-methods evaluation of a social franchise programme in India, which included a telemedical intervention, found that poor communication lines led to women reporting negative experiences of care [34]. Among women who accessed caesarean services in Dhaka, Bangladesh, emotionally negative experiences during childbirth were characterised by provider interactions in which women reported “bhoy” [fear], lack of friendliness, and an absence of reassurance from their doctors [45].

Interactions with health care providers and other staff members also influenced women’s care seeking decisions, particularly the experiences of dis/respectful care and dignity. These interactions shaped where women sought out care, illustrating that real and perceived experiences can significantly influence care trajectories. Women attending private facilities in Nairobi, Kenya cited real and perceived negative treatment from staff, including abuse and ostracism, as a reason to avoid public facilities [35]. As one respondent reported, “Personally, I prefer private hospitals because they are not abusive, and they have to talk to you nicely because they know your money is what has brought you there.” [35] For health care services that might carry potential stigma, like abortion, the experience of non-judgemental care from health care providers influenced women’s decisions to seek abortion-related care in private facilities in Bihar, India [36]. Positive provider interactions, including feeling valued and being treated with dignity, led to women attending the Casa Materna in Guatemala to report positive experiences of care (Schooley 2009). Similarly, among married women seeking care in Tamil Nadu, India, having care needs respected was linked to higher rates of satisfaction (Audinarayana 2008).

Respondents in multiple studies reported privacy and confidentiality of services as essential components of experience of care. A survey of women receiving first-tier public and private antenatal care in Tanzania emphasised the relationship between privacy and positive experiences of care in both facility models [52]. Two different studies used privacy as an indicator of experience of care. In Nepal, reproductive health franchises used privacy as an indicator to measure success of an intervention to improve quality [53]. In Uganda, an intervention to improve quality of care provided by private sector midwives included privacy as a critical area of focus [49].

Within the user-centred health systems domain of experience of care, evidence from India indicated room for improvement. Clinical observations of 275 mother-neonate pairs at 26 public and private hospitals in Uttar Pradesh, India, found that health care workers avoided harmful or unnecessary interventions for the mother in only 6.6% of observations in public facilities and 1.5% of observations in private facilities [24]. Health care workers avoided harmful or unnecessary interventions for the neonate in 33.2% of observations in public facilities and 39.0% of observations in private facilities [24]. These differences between facilities were not statistically significant for mothers or neonates.

Effective quality of care also emerged as a sizeable clinical component of experience of care within the user-centred health systems domain. The provision of care that is effective, or perceived to be effective, shaped the expectations and satisfaction of care-seekers, including the provision of quality counselling, provision of essential equipment, and choice to avoid overmedicalized care. Women valued knowledge of available options and choices. In Dhaka, Bangladesh, women who had caesareans in the private sector reported that a lack of medical counselling, overmedicalization of birth, and presentation of birth options prior to care had negative implications: “I think, the doctor did not say anything, because it involves a lot of money. In private clinics, there is hardly any normal delivery. Now I think, if I had a normal delivery, it would have been better.” [45] Observations of providers in private abortion clinics in Nepal showed that women had positive experiences of the private counselling services due to the counselling being offered in a separate space by a trained counsellor [38]. In Uganda, data from a pre-post-test quasi-experimental study of quality improvement in antenatal care used an eight-scale antenatal care counselling score to measure quality; the study revealed differences in experiences of care among women of different ages [54]. The results indicated that antenatal counselling scores were lower amongst older women, leading the researchers to surmise that older women may have had prior pregnancies and thus found counselling less important.

In addition to linking to experience of care, interpersonal communications and staff treatment also impacted satisfaction with care. In an intervention aimed at improving quality of post-abortion family planning provision, women reported higher satisfaction due to their improved experiences of counselling and time spent with provider: 69% of women reported being given clear care instructions post-intervention compared to 58% of women at baseline [55]. In public-private partnership facilities in a district hospital in Southern India, waiting time for treatment and the manner of other support staff [compared to physician or nurse] were the most significantly associated factors with satisfaction among parents of admitted children [56].

#### Timeliness as an essential component of care

Numerous studies used “time” as a measure corresponding to user-centred health system indicators and also to broader aspects of experiences of care. Timeliness was conceptualised differently across studies, including the time that mothers, newborns, and children spent waiting and receiving care in facilities; the associated time costs in accessing care (e.g., transportation times); and time-based accessibility (e.g., facility opening hours).

Wait times directly affected experiences of care with longer waiting times resulting in poorer experiences of care. In Uttar Pradesh, India, women reported waiting “a number of hours” before being able to attend a telemedical consultation with a health care worker, leading to significantly negative experiences of care [34]. In private, not-for-profit health care centres offering MNCH services in Uganda, a controlled trial found that long wait times resulted in major dissatisfaction among care seekers [57].

Conversely, shorter wait times generally led to higher satisfaction and positive experiences of care. Women accessing abortion services in Istanbul, Turkey, reported positive experiences of short wait times; most women obtained an abortion within 1 to 10 days, whereas women seeking abortions from a public hospital had to wait at least a week [41]. In western Kenya, an intervention aimed at increasing post-abortion contraception uptake among women in private sector clinics emphasised time taken with clients as an essential indicator successful of quality improvement [55]. Even when waiting times were shorter in private facilities than in public facilities, clients were not always satisfied. A comparative study in Benin and Malawi illustrated that satisfaction with care can be linked to one’s expectations of a facility. In the study, 20% of clients in Benin and 17% of clients in Malawi were dissatisfied with median waiting times that ranged from 20 to 38 min and 5–13 min, respectively [58].

Insufficient time with health providers generally resulted in poorer experiences of care and even poorer health outcomes. Not being allowed “enough time” with doctors negatively impacted women’s experiences, as reported by women who had caesarean sections in Dhaka, Bangladesh [45]. These women reported time constraints in public hospitals as a reason to seek private services. In a study of quality of information communication during antenatal care visits in Bahawalpur, Pakistan, researchers used time spent with women as a core indicator and found that providers in private hospitals spent an average of 5 to 8 min per woman compared to 4 min in public hospitals [37]. Evidence among women accessing maternal health services in India indicated that limited time with staff during treatment and lack of decision-making power were linked to worse health outcomes [39]. The time a person is able to spend with a health provider intersects two components of experience of care—timeliness and staff interactions—and highlights the interconnected nature of the components that comprise experience of care.

A facility’s opening hours, an important component of “timeliness,” also influenced decisions to seek care in the private sector. Women in Kenya reported better experiences accessing MNCH services in a private facility “since it operates all the time”; in addition, the facility’s hours influenced women’s decision-making about where to obtain services [35].

#### Contextual inequalities

Inequalities were present in women’s experiences accessing their choice of care as well as women’s experiences receiving care. These inequalities reflected contextual structural hierarchies of discrimination, with studies reporting differences in experience due to income, ethnicity (particularly for Indigenous women), and mobile/internet connectivity. Inequalities primarily occurred at point of access. In private prenatal clinics in Mexico, lower-income and Indigenous women were treated differently in private facilities than higher-income and non-Indigenous women, whereas in public facilities there was no difference [59]. This treatment led to a significant difference in the quality of care provided; people treated worse by health providers received treatment associated with worse quality. A study of a social franchise programme, which included a telemedical intervention, indicated that structural inequalities like access to quality telecommunications meant that women with worse connections had worse experiences of accessing care [34].

Most research focused on affordability and its associated impact on experiences of private health care. Affordability is an essential component of user-centred health systems and was a consistent barrier for women seeking care in private facilities. Experiences of care among women seeking caesarean sections in Dhaka, Bangladesh were better in private than public facilities; private facilities were only available to higher income women who had the necessary resources to exercise choice [45]. Studies examining satisfaction of care also included evidence of costs that varied widely within health systems [57]. Similar findings occurred at abortion clinics in Istanbul, Turkey, where costs of care reportedly ranged from nothing in public facilities to $375 at facilities operated by NGOs, leading women to say, “if you’re rich in Istanbul, you have no problem [accessing care].” [41].

The experiences of women in informal settlements in Nairobi, Kenya who sought MNCH services highlighted the extent of this variation in cost. While some women could take advantage of payment schemes in private hospitals (e.g., pay after care received), others found private health care prohibitively expensive [35]. An analysis of pricing systems of maternal health care in urban India highlighted the impact of costs on choice of care. Certain procedures were too expensive, and deliveries using instruments cost twice the median household income for women in India [39]. Affordability has important implications for service use. In Uganda, follow-up surveys with women attending private clinics found that women were 1.8 times as likely to return to the same clinic if they had experienced “fair charges” [49].

## Discussion

By systematically reviewing the evidence of mothers’, newborns’, and children’s experiences of care in the private sector, this article provides insights into best practices for delivering high quality experiences of care. The findings illustrate the importance of interpersonal relationships with health care workers, in particular staff friendliness, positive attitudes including non-judgement, time spent listening to women, and responsive counselling. The relationship between mothers, newborns, and children and health care workers is essential and cannot be underestimated, as it impacts person-centred care, timeliness, and equity. Timeliness of care, privacy, and affordability were also important aspects of women’s experiences of care in the private sector and by extension, women’s health outcomes. These components of positive experiences of care emerged as noteworthy in both quantitative and qualitative studies. Researchers frequently mixed experience of care and satisfaction with care in their studies, emphasising the complexity of these measures and their variation across contexts as well as their subjective nature amongst people seeking care.

Beyond “affordability” as a socioeconomic measure of inequality, there is a paucity of evidence on inequalities and experiences of private health care among women, children, and newborns. Parents and caregivers may or may not judge care provided to themselves and that provided to their children differently (e.g., a caregiver may be prepared to wait longer for care for themselves than their child, a caregiver may be willing to pay more for care for their children than themselves). Experiences in private facilities raise important questions on the affordability and by extension, the choice of care that women, children, and newborns may obtain. Compared to public health systems, private health systems may both directly and indirectly exacerbate socio-demographic inequalities. People seeking private health care may experience prohibitive costs at point of care; Indigenous and lower-income women may experience lower quality services. These inequalities and the continued marginalisation of certain populations have been identified as challenges across mixed health systems, regardless of the sector (public or private) in which people seek and receive care.

The context of private sector delivery of quality MNCH care is essential to consider. Studies highlight the varied and complex roles that the private sector has in different settings, for example, facilitating MNCH care delivery in areas where public sector infrastructure and capacity is weaker [60] or providing alternative options for care where public health facilities exist [21]. Whilst some of the included studies linked fewer people seeking care in certain types of private facilities with components of experience of care (e.g., lower waiting times, more time spent with health care providers, higher expectations of quality due to higher costs of care) [61], this linkage was not universal. To better understand the factors influencing higher scores of experience of care in certain private health facilities, it is necessary to examine the context of the private health sector, including evidence on the accessibility and coverage of private facilities as well as disaggregation by the type of private provider.

This systematic review contributes to previous reviews on the public and private health sectors in low- and middle-income countries [62, 63]. It complements findings from Basu et al. that timeliness and staff interactions were important components of experience of care [62] and findings from Berendes et al. that the private sector is possibly more client oriented than the public sector [63]. As this systematic review focused on private sector delivery of quality MNCH care, it corroborates that timeliness and staff interactions were major factors in mothers’, newborns’, and children’s experiences of quality care in private medical facilities. Included comparative studies of public and private health providers offer useful suggestions for policymakers, including the possible need to regulate the provision of care and to improve specific elements of experiences that are important, in order to ensure positive service provision of quality experiences of care across mixed health systems.

Due to language restrictions, relevant studies may have been excluded from this systematic review, particularly studies from Latin American and the Caribbean. We were also unable to locate 108 texts, despite our best efforts to locate authors and library assistance. The majority of these missing texts appear to be grey literature, particularly abstracts from the Popline database that closed in September 2019. Moreover, publication bias, particular in intervention research, may have resulted in an increased proportion of negative or neutral findings being rejected for publication [64]. This potential source of bias has important implications on generalizability of claims in this article. Furthermore, this systematic review focused on experience of receiving care in the private sector, but we must also acknowledge the importance of private providers’ experiences implementing interventions to improve quality of care in the private sector [65].

In conducting this systematic review, we observed that studies and reports largely do not consider the delivery of MNCH care in the mixed health system. Thus, the design, analysis, and reporting of studies are often not organized in a way that captures mixed health system data or presents disaggregated findings. As a result, this oversight leads to cherry picking of findings or conclusions, poor comprehension of the actual situation, and at times, the politicization of private sector service delivery.

## Conclusions

Insights from this systematic review confirm that experience of care is fundamental for people seeking quality MNCH care in the private sector. The private sector is varied, complex, and context specific. While inaccessibility amongst some private services allows for privileged experiences of care, this is not inevitable. Meaningful engagement with, and regulation of, private sector service delivery can support efforts to achieve universal health coverage with quality. Of course, providing quality MNCH care and ensuring positive experiences of care in mixed health systems require the existence of basic prerequisites like the availability of clean water, sanitation, and hygiene; essential equipment; medications; and enabling environments for health care workers.

Policymakers, programme implementers, and private sector stakeholders must recognise that experience of care can be a stronger determining factor in health-related decision making than the quality of care available in public and private facilities. In addition to influencing care outcomes, experiences of care amongst mothers, newborns, and children influence their future decision-making and choice of both health providers and health facilities. Poor experiences of care lead people and their caregivers to seek care elsewhere, thus encouraging private providers to prioritise experience of care to ensure market competitiveness and future revenue.

Findings from this systematic review support recommendations aimed at strengthening the evidence base on experiences of private sector MNCH care and recommendations for improving experiences of private sector MNCH care. In order to better understand experiences of quality MNCH care within the private health sector, the following recommendations are made for researchers:

- Experience of care is frequently only a small component of studies on quality of care. Additional research should centre experience of care as a key focus, explore the relationship between experience of private sector quality care and socioeconomic inequalities, and fill in gaps around mothers’ experiences of private health care for childbirth.
- Experience of care should not be an outcome limited to adults. The experiences of children, newborns, and parents or carers seeking care for their children can also add value to studies investigating quality of care.
- Establishing criteria for understanding experience of care would help reduce variations within qualitative and quantitative data. Satisfaction and experience of care are important but separate constructs. Criteria more clearly presenting the differences between experience of care and satisfaction with care are required, as is the centring of inequalities in experiences of care.
- This systematic review excluded 64 studies that presented aggregated findings on the public and private sectors and 45 studies that did not specify whether the health facilities were public and/or private. To better understand experiences of care in the private sector, we encourage researchers to specify what types of health facilities are included in the study and to disaggregate their data on the public and private sectors when conducting analyses. Had we been able to draw upon this work and knowledge, we might have been able to generate more conclusive evidence on experiences of quality MNCH care provided by the private and public sectors.
- The private sector is heterogenous, so having additional details about the types of private facilities included in a study (e.g., self-financing, faith-based, NGO) could show valuable differences.

In order to improve experiences of MNCH care within the private health sector, the following recommendations are made for policymakers and programme implementers:

- Patients seeking MNCH care may switch between the public and private sectors, obtaining private antenatal care but choosing to deliver in a public facility, for example. Improving experience of care requires approaching the entire mixed health system and also strengthening case referrals between the public and private sectors.
- In certain settings, a lack of economic incentives to establish and operate private health facilities delivering MNCH care in rural areas means that private health facilities are likelier to deliver MNCH care in urban areas and to people from higher socioeconomic groups. Expanding access to low-interest loans and providing economic incentives to private health facilities can help facilitate the entry of private MNCH providers in hard-to-reach and rural areas as well as amongst lower socioeconomic groups, thus expanding access to affordable quality care and helping deliver universal health coverage.
- Even if mothers, newborns, and children report positive experiences of care, poor dissemination of updated guidelines and standards to private health providers means that the quality of MNCH care delivered may not always align with national standards. Greater dissemination and outreach to private facilities and providers is warranted as is the inclusion of stakeholders from the private sector in developing national policies, standards, and strategies. Additional resources and easier access to financing can also help facilitate greater compliance with national quality standards.
- Comparative studies of experiences of quality MNCH care in public and private health facilities provide opportunities for shared learning between facilities; however, this cross-learning is rarely instutionalised. We encourage Ministries of Health to strengthen and operationalise a mechanism for public-private dialogue in order to foster relationships, create open and transparent communication, and co-develop and co-implement an agenda to strengthen quality of MNCH care.

## Supplementary Information

**Additional file 1.**

**Additional file 2.**

## Data Availability

The data extraction workbook is available on request from SRL (lattofs@who.int).

## Abbreviations

NGO: Non-governmental organisation
MNCH: Maternal, newborn, and child health
WHO: World Health Organization
