# Supplementary material for: Experiences of private sector quality care amongst mothers, newborns, and children in low- and middle-income countries: a systematic review
Source: BMC Health Serv Res. 2021 Dec 6;21:1311. doi: 10.1186/s12913-021-06905-3 (PMC8647361; doi:10.1186/s12913-021-06905-3)
Supplement: Supplementary file 1 — Additional file 1. [file 12913_2021_6905_MOESM1_ESM.docx]

**Supplementary Annex 1: Summary table of included studies reporting outcome data on experience of care (n=45)**

| **Author, year [country]** | **Aim/objective(s)** | **Setting and population** | **Intervention description** | **Study design** | **Summary of findings on experience of care** | **Quality** |
| --- | --- | --- | --- | --- | --- | --- |
| (Agha, Balal et al. 2004) [Uganda] | To assess the impact of a microfinance intervention with private sector midwives | Private midwives who were members of the Uganda Private Midwives Association for intervention (n=15) and control (n=7) | Loans provided to midwives on a revolving basis, with 15 midwives taking an average loan of $454, 11 repaying and taking second loans averaging $742.  Training of basic business skills was provided to loan recipients via the National Smallholder Business Center.  Follow up assessments of midwife performances were conducted | Quasi experimental | Intervention clients were significantly more likely to report at follow up that privacy was the reason for their preference of intervention clinics. This trend was not significant for comparison clinics. There was no net effect of the intervention on good handling of clients, clinic accessibility, good physical outlook of the clinic, or on the range of services provided.  Clients at intervention clinics were 1.8 times more likely than clients at comparison clinics to report that they always visited the clinic. Clients who reported fair charges as their reason for visiting were 1.8 times more likely to report they always visited the clinics. Clients who reported good handling of clients as their reason for visiting were 1.8 times more likely to report they always visited the clinic. Clients who reported privacy as their reason for visiting were 1.5 time more likely to report they always visited the clinic. | Weak |
| (Agha, Karim et al. 2003) [Nepal] | To examine the performance of a nurse and paramedic network, established to increase quality and utilization of reproductive healthcare services in Nepal | 64 private nurses and paramedics | Network members were provided 7 days of training on reproductive health, including family planning, A subset received additional intrauterine device (IUD) training. Intervention recipients received marketing and promotional training, referral linkages, and a quality monitor who visited monthly. | Quasi experimental | The intervention significantly increased the percentage of clients who were “very satisfied” with the cleanliness and availability of essential equipment, neither of which changed in the control clinics. In addition, intervention clients reported better satisfaction with privacy after the intervention, not reflect in follow-ups in control clinics. Neither intervention nor control clients reported significant changes in satisfaction for client handling or service charges.  Increases in satisfaction were associated with increased odds of making return visits to the intervention clinics. Unobserved clinic-level influence on return visits was mainly explained by perceived clinic quality. | Weak |
| (Akhter and Schech 2018) [Bangladesh] | To understand why caesarean sections have become widespread among wealthier women in Dhaka | 30 women who had given birth in the preceding five years | Delivery of quality care by the private sector | Qualitative: in-depth interviews | Two key factors were identified as contributing to complex childbirth care experiences: 1) limited information about childbirth among women; and 2) unequal client-service provider relationships, which subordinated women to medical experts. | Weak |
| (Akwara, Alayon et al. 2003) [Uganda] | To measure changes in reproductive, maternal, and child health knowledge and behaviour in Delivery of Improved Services for Health (DISH) project districts | Health care facilities in 12 districts of Uganda | The study evaluates four primary interventions:  1. Yellow Star Program: partnered with Ministry of Health Quality Assurance Department and Health Promotion and Education Decisions to design 35 basic standards of high equality health services.  2. Adolescent-Friendly Reproductive Health Services: expansion of services to 34 health centres in 12 districts, including marketing and publicity services  3. Long-Term and Permanent Methods Marketing and Services: Staff training in seven hospitals to provide routine tubal ligation, vasectomy and Norplant services, as well as midwives and clinical officers to remove Norplant and counsel on other services.  4. Safe Motherhood Strategy: A radio and print campaign, as well as birth-planning cards, distributed to promote quality maternal care. A self-instructional manual for healthcare providers, community discussions by midwives, and community reasons persons to follow up people after antenatal care (ANC) clients. | Survey and facility observations | One-third of consultations were rated excellent or acceptable for counselling and education. Within government facilities, trained providers were significantly more likely to score excellent or acceptable: only 1% of ANC visits in these facilities were by untrained providers rated acceptable and none were rated excellent, compared to 19% of visits by trained providers rated acceptable or excellent. | Weak |
| (Alcock, Das et al. 2015) [India] | To quantify the pattern, determinants, and choice of maternity care provider at the health facility level in the public and private sectors in Mumbai’s informal urban settlements | Survey of married women aged 15-49 (n=3,848) and interviews with married women over 18 (n=78) in two municipal wards of Mumbai | Delivery of quality care by the private sector | Mixed-methods | Experience of care was associated with avoidance and discontinuation of certain care pathways. One respondent ceased care at a public hospital, reporting being “made to run around” while attempting to register. One respondent was afraid of being forced to undergo a caesarean section and so opted for home birth. | Weak |
| (Alireza, Farahbakhsh et al. 2006) [Iran] | To implement and evaluate the efficacy of a new model for transferring state service delivery governance to nongovernmental groups and studying if they can be efficient in the field of child and maternity health services compared to public health centres | 9 cooperative health centres and 18 public health centres | Advertised for applications from healthcare teams to transfer from health service delivery to a cooperative health centre (CHC). CHCs received periodic visits and delivery protocols designed by Ministry of Health, and per-capita payments for each service package through contracts with provincial health departments. | Case-control | Cooperative health centres had significantly better indices than public health centres on: timely availability of services, cleanliness of health centres, giving sufficient and suitable education, dedicating sufficient time for each service, acceptable waiting time for receiving services, and friendly attitude of personnel. | Weak |
| (Allam, Oruganti et al. 2016) [India] | To evaluate HIV/AIDs services as part of the APAIDSON program, India | 115 patients accessing HIV testing and counselling and 115 people living with HIV in patient wards | Delivery of quality care by the private sector | Mixed-methods | Measures taken included reducing stigma and discrimination in the workplace (through training and sensitization programmes), guidelines and protocol implementation to reduce stigma and discrimination, prevention of segregation e.g. preventing separation of beds or identification marks, staff compensation and encouragement of universal precautions. Beneficiaries reported overall satisfaction as high. | Weak* |
| (Alyahya, Khader et al. 2019) [Jordan] | To explore the quality of maternal-foetal and newborn care in ANC, delivery, and postnatal care (PNC) services to better understand gaps in completeness of care | 52 pregnant and postpartum women receiving maternal childcare | Delivery of quality care by the private sector | Qualitative: focus group discussions | There were mixed preferences for private and public antenatal care services. Respondents perceived that quality of the equipment and the devices in the private sector would be better than the public sector.  Women cited lack of trust in healthcare professionals’ skills and abilities, privacy concerns, and less ‘centred care’ as reasons for delivery care services in the public sector being suboptimal.  Some respondents perceived that interpersonal and communication skills would be better among private sector obstetricians, and that if they used the private sector, they could ask for a woman to provide their care. Longer waiting times discouraged antenatal follow-up in public facilities. | Moderate |
| (Angeles, Hutchinson et al. 2003) [Bangladesh] | To collect information on and monitor changes in the United States Agency for International Development (USAID) performance indicators since the Rural Service Delivery Partnership (RSDP) Baseline Survey and to evaluate the impact of the RSDP programme on the health of the project population | 9768 women ever married aged 10 to 49 years in RSDP program areas and 3,176 from comparison, non-programme areas | Delivery of quality care by the private sector | Cohort analytic study | Approximately 15% of users of RSDP clinics reported that services were free of charge and 65% that cost of treatment was reasonable. The mean waiting time was 18.5 mins, 44.5% of users did not have to wait.  The majority of users rated staff behaviour (99.3%) and quality of services (98.7%) as good or very good and most (99.4%) would recommend services to others. | Moderate |
| (Angeles, Lance et al. 2005) [Bangladesh] | To collect information about knowledge, awareness, and the use of services related to family planning and maternal and child health through the non-governmental organization (NGO) Service Delivery Program and alternatives | 5,691 women ever married aged 10 to 49 years in NGO Service Delivery Program areas and 4,201 from comparison, non-programme areas | Delivery of quality care by the private sector | Cohort analytic study | 93.7% of users of NSDP satellite clinics reported that staff spent enough time with them, 85.7% that they were spoken to nicely and 95.8% that there was enough attention to their needs. The mean waiting time was 14.3. minutes and the mean travel time was 7.2 minutes.  95.1% of NSDP clinic users reported staff spent enough time with them, 90.3% that they were spoken to nicely and 97.6% that enough attention was paid to their needs. Mean travel time was 13.1 minutes and wait time was 20.8 minutes.  There was little variation in perceptions of quality of treatment between users of NSDP, government or other services. | Weak |
| (Angeles, Lance et al. 2006) [Bangladesh] | To measure changes in the USAID performance indicators for the NGO Service Delivery Program since mid-project evaluations in 2003. | 7,652 women ever married aged 10 to 49 years in NGO Service Delivery Program areas and 4,418 from comparison, non-programme areas | Delivery of quality care by the private sector | Cohort analytic study | Overall satisfaction for NSDP services was high – almost all users reported providers spent enough time with them, had good interpersonal skills and paid them attention. NSDP providers rated slightly higher than public sector services in all measures of quality. | Moderate |
| (Audinarayana 2008) [India] | To review the use of private and public healthcare services among married women in Tamil Nadu through a review of data | Currently married women aged 15-44 in Tamil Nadu | Delivery of quality care by the private sector | Data and literature review | Studies show that satisfaction with services was high across sectors, with the private sector having 98% satisfaction rates and public sector 85%. More women reported that staff talked to them nicely and respected their needs for privacy in private than public facilities.  Experiences of care between public and private facilities varied across districts and length of waiting time and quality of services were cited by women in some studies as reasons to use private services as opposed to government. | Moderate |
| (Babirye, Engebretsen et al. 2014) [Uganda] | To examine services and community sub-systems relating to an immunisation programme in urban settings | 821 caretakers of children aged 10-23 months in Kampala, 58 women and 15 men in focus group discussions (FGDs), and 5 healthcare providers in key informant interviews | Delivery of quality care by the private sector | Mixed-methods | Findings differed between data collection tools. Women in FGDs reported a preference for public facilities as they perceived that service providers were better qualified.  Delays and waiting times were most frequently associated with public facilities. Women reported that these delays could mean that they were not able to receive immunisation treatment on their visits. | Weak |
| (Bakibinga, Ziraba et al. 2016) [Kenya] | To examine the perspectives of women and community health volunteers (CHVs) regarding the use and quality of maternal and child health services in public and private facilities | 849 women and girls aged 12-49  936 children [via caregivers] from Korogocho and Viwandani informal settlements, Nairobi | The intervention was entitled ‘Partnership on Maternal, Newborn and Child Health’, and included: infrastructural upgrade of selected Private Not-For-Profit (PNFP) health facilities operating in two informal settlements; building capacity for both health care providers and the sub–County Health Management Teams in Ruaraka, Kasarani and Makadara sub-counties of Nairobi, where Korogocho and Viwandani, respectively, are located; and facilitating provision of supportive supervision by the local district health authorities that are forming networks of CHVs to create demand for the health services. | Mixed-methods | Quality of services was measured in the time spent at the facility before services are received, availability of drugs and in the attitudes of clinicians. Respondents indicated that private facilities did better in these measures than public.  Other results are mixed. Respondents indicated that they felt that quality of staff training was inadequate in private facilities compared to public, whilst some felt the quality of interpersonal care was better in private than public.  Accessibility was one of the major reasons for preference for private facilities for maternity services, relating to service times and opening hours. | Moderate |
| (Baliga, Ravikiran et al. 2016) [India] | To understand perceived quality of care among parents and children admitted to two medical college hospitals – one run with private partnership models and one operated by the government | Parents of patients in a public private partnership hospital (n=580) and a public health hospital (n=461) in southern India | Delivery of quality care by the private sector | Regression analysis | In the public-private-partnership hospital, duration of time spent waiting and the manner of ‘other’ support staff were significantly associated with overall parental satisfaction, whilst in the government hospital counselling regarding treatment was a significant positive factor for satisfaction.  When controlling for age-group, sex, maternal education level, type of family, hospital-stay and socioeconomic class, the parents of inpatients in the PPP model hospital were significantly more satisfied than those in the government hospital. | Weak |
| (Banerjee, Andersen et al. 2015) [India] | To describe the implementation of the Yukti Yojana programme to expand availability of safe abortion services in Bihar, India | 48 in-depth interviews of providers and key stakeholders and 16 facility evaluations | The programme was advertised through media and aimed to accredit private sector health facilities to provide abortion-related services free of charge.  A technical advisory group monitored quality of the programme, including an NGO contracted to monitory quality of services, and an organisation to provide independent data collection.  Facilities that were given accreditation by a committee were contracted and reimbursed for provided abortion services. | Descriptive analysis | Satisfaction levels included abortion providers non-judgemental attitudes and behaviours. Less than half of women receiving abortion services (46%) reported very high levels of satisfaction compared to moderate (44%) and low level of satisfaction (10%). | Moderate |
| (Barber, Bertozzi et al. 2007) [Mexico] | To evaluate prenatal care quality in rural Mexican communities | 3533 women who reported they received prenatal services between 1997-2003 | Delivery of quality care by the private sector | Regression analysis | People most economically disadvantaged received below average care in the private sector and above average care in the public sector. There were significant differences in the adjusted means of quality of care between indigenous and non-indigenous women who sought care in private clinics but not in public clinics. | Strong |
| (Béhague, Gonçalves et al. 2002) [Brazil] | To explore how healthcare users, professionals, and local politicians viewed and reacted to the changes from the 1987 constitutional revision, including the expansion of the primary health care (PHC) network | 80 women who had given birth in 1993 and 26 healthcare providers | Delivery of quality care by the private sector | Mixed-methods | In Pelotas, users tended to have more positive experiences of private care despite public primary care having higher quality care provision.  Despite using public primary healthcare, some women paid for private consultations as a form of “security” against perceptions of bad quality care, or sought initial care elsewhere and used PHC for free services, which led to negative experiences among PHC staff. | Weak |
| (Boller, Wyss et al. 2003) [Tanzania] | To compare the quality of antenatal care offered by public and private providers | 166 women attending public health facilities  188 women attending private health facilities | Delivery of quality care by the private sector | Regression analysis | Seats were available for 89% of women attending public facilities and 93% attending private facilities. Privacy of the consultations was observed in 81% of consultations in the public sector and 99% in the private sector. Women were invited to talk about their medical concerns in 71% of consultations in public facilities and 81% in private facilities. The general history of pregnant women was taken in 35% of consultations in public sector and 49% in the private sector. | Weak |
| (Chirdan, Lar et al. 2013) [Nigeria] | To quantify differences in the quality of maternal health services at public and private health centres and to assess how these quality differentials impact upon maternal health clients’ satisfaction | Women accessing maternal health services whose children came from first dose of diphtheria, pertussis, and tetanus (DPT) vaccine in private facilities (n=97) and in public facilities (n=204) | Delivery of quality care by the private sector | Cross-sectional | They found that between private and public health facilities there were generally similar trends in satisfaction when measuring waiting times, waiting lounge conditions, cleanliness, and services. | Weak |
| (d'Orsi, Bruggemann et al. 2014) [Brazil] | To identify factors associated with women’s satisfaction regarding the relationship they develop with health practitioners during hospital stay and childbirth | 23,523 women interviewed six months before birth and 15,688 followed up 12 months after birth | Delivery of quality care by the private sector | Regression analysis | Higher proportions of verbal, physical and psychological abuse were reported among non-white women, of lower school education, aged between 20-34 years, of the Northern Region, with vaginal delivery, no companion during hospital stay, who were attended by public sector care workers.  In the adjusted analysis of the Southern Region, delivering in the private sector was associated with lesser chance of suffering abuse.  Satisfaction was significantly higher among women whose childbirth was paid by the private sector. | Weak |
| (Danel and Forgia 2005) [Guatemala] | To assess the performance of Guatemala’s programme to extend coverage of basic health services and to determine the relative economic efficiency of different service delivery models | National with a target population of children under 5 and prenatal care seekers. | Delivery of quality care by the private sector | Regression analysis | Community centres in both mixed and direct provider communities scored well for friendliness of care and waiting times compared to traditional health posts. Satisfaction rates were overall generally high and the perception of problem resolution high across all provider types. | Weak |
| (De Savigny, Mayombana et al. 2004) [Tanzania] | To analyse care-seeking events in a large series of malaria deaths recorded in the course of longitudinal demographic surveillance | Demographic surveillance of all residents in the Rufiji District | Delivery of quality care by the private sector | Mixed-methods | A respondent reported preferring the hospital services due to experiencing better, more reliable quality care for her child than traditional healers. | Weak |
| (Diamond-Smith, Sudhinaraset et al. 2016) [Kenya and Namibia] | To describe the clinic quality of facilities in Kenya and Namibia, the perceived quality of patients exiting facilities, and determine if perceived quality is predictive of clinical quality | National Service Provision Assessment data linked to the Demographic and Health Survey | Delivery of quality care by the private sector | Regression analysis | At exit, clients from private ANC facilities gave significantly lower complaint scores than those from public facilities in Kenya – this was not significant in Namibia. This was despite private facilities having lower quality of care in general. | Moderate |
| (Do and Agha 2009) [Uganda] | To evaluate the quality of three types of services – antenatal care, family planning, and postnatal care | Private midwives across Kampala and three regions (Central, Eastern and Western) were put into three intervention groups: A (n=85), B (n=89), and a comparison group (n=74) | A quality improvement packaged included: a form to review service statistics, a provider self-assessment tool, a linked action plan, and a tool to enable supervisor solutions. Intervention Group A were midwives who received one-day training on how to use the tool, supervisors were not trained. Intervention Group B consisted of midwives and supervisor training. | Pre-post-test quasi-experimental | Older clients had significantly lower counselling scores than their younger counterparts. In the follow-up sample, pregnant women with secondary education or higher received technical services of higher-than-average quality compared to their peers.  At follow-up, employed clients received technical services of a significantly lower-than-average quality compared to clients not employed. | Weak |
| (Duggal and Ramachandran 2004) [India] | To synthesise findings as part of the Abortion Assessment Project | Policy documents from multicentre facility surveys in Tamil Nadu, Karnataka, Andhra Pradesh, Maharashtra, Gujarat, and Haryana | Delivery of quality care by the private sector | Policy analysis | Private facilities were perceived as better than public due to less time to obtain abortions, better equipment and longer rest periods after receiving care, and greater confidentiality. | Weak* |
| (Health Partners International and Montrose 2015) [Uganda] | To summarise the issues around quality of care noted throughout the life of the Northern Uganda Health programme and provide lessons for policy makers | 31 faith-based, private not-for-profit healthcare providers | Health facilities received a Quarterly Quality Assessment (QQA) and an annual assessment of staffing to assess and monitor the quality of care provided. Direct Client Verification of care received was also conducted to compare different services. | Multivariate analysis | 78% of clients contacted reported being satisfied with care due to comprehensive services and positive staff attitudes. Factors relating to dissatisfaction included long waiting times, overcrowding of facilities, lack of medicines and poor facility upkeep. | Weak* |
| (Hulton, Matthews et al. 2007) [India] | To present evidence of a situation analysis of quality of care within institutional maternity services | 4 public providers of maternity health services | Delivery of quality care by the private sector | Case study analysis | Women’s experienced were generally better at private than public facilities, reporting less crowded spaces and better cleanliness.  Similar proportions of women (approximately three quarters) in private and public facilities did not have their labour and childbirth care explained to them. One in four women across facility types reporting feeling isolated and worried.  Costs in private facilities could be twice the median monthly income and the private facility model may encourage the use of more expensive delivery methods. | Weak |
| (Jallow, Chou et al. 2012) [the Gambia] | To assess women’s preferences and perceptions of antenatal healthcare services in public and private healthcare facilities | Women attending ANC in public (n=264) and private (n=238) facilities in the Western region | Delivery of quality care by the private sector | Cross-sectional | Less than half of the total sample received care information and felt reassured; significantly more women in private clinics felt reassured compared with their public-facility counterparts.  More than 70% of women across facility type were satisfied with the number of ANC check-ups they received.  Women attending public clinics received significantly lower levels of provider attention than women in private clinics, with the mean waiting time significantly higher and more women in the public sector being unhappy with the waiting times. The mean time spent with providers was significantly shorter in public than private clinics (3.7 minutes compared to 6.6 minutes). Women were happier about the facility space and neatness in private clinics and 98.3% were happy with the privacy offered compared to 90.2% in public clinics. | Moderate |
| (Karki, Ojha et al. 2009) [Nepal] | To conduct a baseline survey on existing government approved comprehensive abortion care services | Abortion care services providers in Nepal, including providers from MSI (Marie Stopes International) Choices and Family Planning Association of Nepal (FPAN) | Delivery of quality care by the private sector | Healthcare centre observations | Counselling was considered better at private facilities due to a separate designated space and an appointed, trained counsellor, compared to government owned sites. | Weak* |
| (Kojima, Bristow et al. 2015) [Haiti] | To reintroduce newborn male circumcision (for non-medical reasons) in Haiti | 2 obstetricians and 7 nurses | Staff were trained on the technique, common complications and management, outcome survey data collection. Training included practice surgeries with newborns. Nurses were trained to ensure participant flow, preparation and education. | Descriptive study | One parent reported difficulty with postprocedural care and that they were destressed because of their child’s crying. | Weak |
| (Levin, Munthali et al. 2019) [Benin, Malawi, Georgia] | To understand the role of private sector provision and financing of vaccination services | In Benin, 295 vaccination clients across 35  private for-profit, 9 faith-based, 6, NGO, and 10 public facilities.  In Malawi, 310 vaccination clients across 16 private for-profit, 21 faith-based, 5 NGO, and 11 public facilities.  In Georgia, 301 vaccination clients across 44 private for-profit facilities, 3 private maternity facilities, and 3 private hospitals. | Delivery of quality care by the private sector | Case studies | 88% of clients in Malawi, 90% of clients in Benin, and 97% of clients in Georgia indicated that health workers answered their vaccination queries; however, 13% of clients in Benin and 15% of clients in Malawi were dissatisfied with the amount of explanation provided.  Though waiting times in private facilities were lower than in public health facilities, they were the main course of dissatisfaction. | Weak |
| (MacFarlane, O'Neil et al. 2017) [Turkey] | To document both married and unmarried women’s experiences obtaining abortion services in Istanbul | 14 people who had obtained abortions | Delivery of quality care by the private sector | Qualitative: in-depth interviews and case studies | Women who sought private sector abortions frequently described it as “easy”, with shorter waiting times and time between consultation and receiving care. However, the costs were prohibitive for lower-income women seeking care. | Moderate |
| (Mahar, Kumar et al. 2012) [Pakistan] | To assess the quantity and quality of information, education and communication during antenatal care in public and private hospitals of Bahawalpur | Pregnant women attending obstetric outpatient departments at a private hospital (n=108) and a public hospital (n=108) | Delivery of quality care by the private sector | Cross-sectional | Women spent an average of 3 minutes with healthcare providers in public hospitals and 8 minutes in private hospitals. | Moderate |
| (Penn-Kekana, Powell-Jackson et al. 2018) [India] | To report evaluation findings and assess the impact of the Matrika social franchise programme in Uttar Pradesh | 365 SkyCare providers, 50 SkyHealth centres, 8 franchise clinics, 58 private providers, 50 private facilities, and 2,149 accredited social health activists | The intervention aimed to improve maternal health through reducing deaths from postpartum haemorrhage by establishing the Sky social franchise network. This engaged private providers at three levels:  1. Skycare providers: informal rural healthcare providers, who were trained to encourage women to use the services in the network and facilitate phone consultations.  2. SkyHealth centres: engaged to provide ANC consultation using telemedicine.  3. Franchise Clinics: private hospitals engaged to deliver emergency obstetric care.  Clinical training and regular quality improvement visits were conducted with SkyHealth centres and Franchise Clinics, and village led information activities were designed to engage women. | Mixed-methods | Women had negative experiences with technical complications from the telemedical consultations and struggles in communication and interpersonal dynamics with healthcare workers. Women reported finding it hard to talk to someone they could only see on a screen. | Strong |
| (Rahman, Rob et al. 2009) [Bangladesh] | To develop a voucher distribution system for women using maternal health services, identifying private and NGO facilities to provide quality services, increase capacity of service providers and improve utilisation | A survey of providers from 3 clinics and 23 fieldworkers  A survey of pregnant women/mothers (n=436) and in-depth interviews with women (n=15) | The intervention provided a 3.5-day orientation for service providers, capacity building of service providers and fieldworkers including three-week training in partnership with Population Council, health facility strengthening to enable the provision of quality services, flipcharts and educational materials to create awareness of the intervention activities amongst communities. | Pre-and-post intervention | Of their quality of care indicators, “treatment in a friendly manner” related to experience and saw improvements at endline in both Bausha and Gaznaipur, with overall improvements from 75.3% at baseline to 95.7% at endline. | Weak |
| (Ramachandar and Pelto 2002) [India] | To report on abortion services in the Coimatore district of Tamil Nadu and the role of government village health nurses in assisting women obtaining abortions | 42 village health nurses working in 10 “sterilisation camps” in different primary health centre locations | Delivery of quality care by the private sector | Qualitative | Health authorities, village health nurses and individual women who had abortions reported that preferences for private providers was due to confidentiality and privacy, avoidance of sterilisation as a condition, perception that they would receive better quality care, and more willingness to provide services beyond the 20-week gestational limit | Weak |
| (Schooley, Mundt et al. 2009) [Guatemala] | To document and assess the validity of anecdotal evidence of the quality of care at the Casa Materna, Guatemala | 21 clients and traditional birth attendants at the Casa Materna, 17 female advocates / promoters of Casa Materna services, and 12 male advocates | As part of the Project Concern International / Guatemala Ministry of Health intervention to reduce maternal mortality, the Casa Materna was established to provide specific maternal, newborn, and child health (MNCH) services through trained providers and increased quality care. Community educators were used to encourage women to utilise these services. | Qualitative: in-depth interviews, focus group discussions and case histories | The Casa Materna focused on patient-centred and appropriate care, with women reported they felt valued and treated with dignity. Positive provider support to counteractive isolation or fear resulted in women reporting they felt affection, solidarity and safety within the Casa Materna. | Strong |
| (Sharma, Powell-Jackson et al. 2017) [India] | To describe and investigate the quality of care provided routinely for uncomplicated labour and childbirth in maternity facilities in Uttar Pradesh | 29 private and 30 public maternity facilities in Uttar Pradesh | Delivery of quality care by the private sector | Clinical observations | Clinical observations of 275 mother-neonate pairs at 26 public and private hospitals in Uttar Pradesh, India found that health care workers avoided harmful or unnecessary interventions for the mother in only 6.6% of observations in public facilities and 1.5% of observations in private facilities.  Health care workers avoided harmful or unnecessary interventions for the neonate in 33.2% of observations in public facilities and 39.0% of observations in private facilities. | Weak |
| (SHOPS Program 2018) [Kenya] | To provide an overview of the SHOPS program | Private healthcare providers in Kenya | SHOPS Kenya partnered with two insurers, two providers, and Savannah Informatics Limited, a Kenyan technology firm, to pilot a new electronic data interchange (EDI) for savings and improved efficiencies. | Descriptive statistics and case studies | One respondent reported having to seek care at multiple hospitals, including selling personal belongings and moving to a less expensive house to pay off the medical bills. | Weak* |
| (Sieverding, Briegleb et al. 2015) [Ghana and Kenya] | To understand experiences with clinical social franchising in three large networks affiliated with INGOs in Ghana and Kenya | 23 providers in BlueStar Ghana. 24 providers in Kenyan franchises (10 from Amua, 14 from Tunza)  21 clients from BlueStar, Ghana. 26 clients from franchises in Kenya (7 Amua facilities, 19 Tunza facilities) | Delivery of quality care by the private sector | Qualitative: in depth-interviews | Clients reported high levels of satisfaction at franchised providers, with reasons including: perceived quality of care; how polite, friendly and caring providers and other staff were; short waiting times; facility cleanliness.  Positive relationships between staff and clients led to respondents reporting higher comfort discussing issues and asking questions. | Strong |
| (Turan, Bulut et al. 2006) [Turkey] | To understand the quality of antenatal and childbirth care at three hospitals in Istanbul | 176 antenatal observations: 43 at the Ministry of Health hospital, 48 at the Social Security Organisation, and 75 at the private hospital | Delivery of quality care by the private sector | Mixed-methods | Observers noted that the all-female maternity staff at the private hospital treated women with generally more respect, courtesy and regard for their privacy than staff in the two public hospitals. | Strong |
| (Vora, Saiyed et al. 2018) [India] | To determine the quality of free delivery care and examine the difference between public and accredited private sector facilities | 1,616 women of reproductive age | Delivery of quality care by the private sector | Regression analysis | Most women (96%) reported obtaining first examination within an hour of reaching the hospital, with 2% reporting that staff shouted, abused, or hit them during delivery. Only 19% of women reported having procedures explained before they were performed, 38% of women reported receiving explanations sometimes, 39% of women reported receiving little to no explanation, and 3% of women did not know. | Weak |
| (Wendot, Scott et al. 2018) [Kenya] | To assess whether post abortion family planning (PAFP) and long-acting reversible contraceptive uptake increased after a quality management intervention in private clinics | 12 clinics providing safe abortion or post abortion care in Western Kenya | A quality management intervention aimed to increase the uptake of highly effective methods of contraception following abortions. Providers attended a one-day orientation covering: a discussion on PAFP, counselling PAFP, training on a job aide given to service providers, values clarification, re-orientation on data reporting for PAFP. A one-page guide to PAFP was provided and a checklist for safe abortion provision. Franchised service providers received monthly supervision visits. | Pre-and-post intervention analysis | At both post-intervention and baseline, women reported high levels of satisfaction with all elements of the service (provider gave clear instructions, made them feel comfortable, took enough time, friendliness and respect from staff, procedure) except cost.  Clarity of instructions and time taken to understand clients were significantly more positive post-intervention compared to baseline. | Weak |
| (Zaidi, Riaz et al. 2015) [Pakistan] | An assessment of contracted facilities and government managed facilities | Two contracted-out rural health centres and four matching government-managed rural health centres | Contracting out of MNCH services since 2008 to national NGOs, including provision of facility-based routine and basic emergency obstetric and newborn care (BEmONC) services | Controlled clinical trial | Contracted rural health centres had higher scores of patient satisfaction than government-managed centres, in relation to satisfaction with services and inclination to delivery at a health facility. | Moderate |

* Two quality assessment tools were used for quantitative (EPHPP 2010) and qualitative research (Walsh and Downe 2006, Solnes Miltenburg, Roggeveen et al. 2013). Both tools were used for mixed-methods studies. Certain studies and reports lacked the information required to use these tools. For example, a paper lacked a clear methodology section required for a comprehensive quality assessment to be made. Due to the missing information, these particular studies have been categorised in the table above as “weak.”

**References**

1. Agha, S., A. Balal and F. Ogojo-Okello (2004). "The impact of a microfinance program on client perceptions of the quality of care provided by private sector midwives in Uganda." Health Services Research **39**(6p2): 2081-2100.

2. Agha, S., A. Karim, A. Balal and S. Sosler (2003). A quasi-experimental study to assess the performance of a reproductive health franchise in Nepal. Country Research Series. Washington, D.C., USAID/Commercial Market Strategies Project**:** 38 p.

3. Akhter, S. and S. Schech (2018). "Choosing caesareans? The perceptions and experiences of childbirth among mothers from higher socio-economic households in Dhaka." Health Care for Women International **39**(11): 1177-1192.

4. Akwara, P., S. Alayon, S. Barry, C. Lettenmaier and V. David (2003). Uganda Delivery of Improved Services for Health (DISH) facility survey 2002, Chapel Hill, North Carolina, University of North Carolina at Chapel Hill, Carolina Population Center, MEASURE Evaluation Project, 2003 May**:** [125] p.

5. Alcock, G., S. Das, N. S. More, K. Hate, S. More, S. Pantvaidya, D. Osrin, T. A. J. Houweling and N. Shah More (2015). "Examining inequalities in uptake of maternal health care and choice of provider in underserved urban areas of Mumbai, India: a mixed methods study." BMC Pregnancy & Childbirth **15**(1): 1-11.

6. Alireza, N., M. Farahbakhsh, K. Ashjaei, T. Djafarsadegh, H. Sadeghi-Bazargani and Z. Akram (2006). "Maternity and Child Health Care Services Delivered by Public Health Centers Compared to Health Cooperatives: Iran`s Experience." Journal of Medical Sciences **6**.

7. Allam, R. R., G. Oruganti, C. Uthappa, N. Simhachalam, J. Rajesh and V. Yeldandi (2016). "APAIDSON program evaluation of the largest private public partnership consortium for HIV/AIDS care and treatment in India." International Journal of Infectious Diseases **45**(SUPPL. 1): 215.

8. Alyahya, M. S., Y. S. Khader, A. Batieha and M. Asad (2019). "The quality of maternal-fetal and newborn care services in Jordan: a qualitative focus group study." BMC Health Serv Res **19**(1): 425.

9. Angeles, G., P. Hutchinson and M. S. Khan (2003). 2001 Rural Service Delivery Partnership Evaluation Survey. Household survey report, Chapel Hill, North Carolina, University of North Carolina at Chapel Hill, Carolina Population Center [CPC], MEASURE Evaluation, 2003 Feb.**:** [182] p.

10. Angeles, G., P. Lance, P. Hutchinson, S. N. Mitra and S. Islam (2005). 2003 Urban NGO Service Delivery Program (NSDP) evaluation survey, [Chapel Hill, North Carolina], University of North Carolina at Chapel Hill, Carolina Population Center [CPC], MEASURE Evaluation, 2005 Mar.**:** [193] p.

11. Angeles, G., P. Lance and M. S. Khan (2006). 2005 Rural NGO Service Delivery Program (NSDP) evaluation survey, Chapel Hill, North Carolina, University of North Carolina at Chapel Hill, Carolina Population Center [CPC], MEASURE Evaluation, 2006 Aug.**:** [186] p.

12. Audinarayana, N. (2008). "Are government health facilities losing their charm in extending maternal and child health care services? A critical review of studies from Tamil Nadu." Health and Population: Perspectives and Issues **31**(4): 234-246.

13. Babirye, J. N., I. M. Engebretsen, E. Rutebemberwa, J. Kiguli and F. Nuwaha (2014). "Urban settings do not ensure access to services: findings from the immunisation programme in Kampala Uganda." BMC Health Serv Res **14**: 111.

14. Bakibinga, P., A. K. Ziraba, R. Ettarh, E. Kamande, T. Egondi and C. Kyobutungi (2016). "Use of private and public health facilities for essential maternal and child health services in Nairobi City informal settlements: Perspectives of women and community health volunteers." African Population Studies **30**(3): 3113-3123.

15. Baliga, B. S., S. R. Ravikiran, S. S. Rao, A. Coutinho and A. Jain (2016). "Public-Private Partnership in Health Care: A Comparative Cross-sectional Study of Perceived Quality of Care Among Parents of Children Admitted in Two Government District-hospitals, Southern India." J Clin Diagn Res **10**(2): Sc05-09.

16. Banerjee, S. K., K. L. Andersen, D. Navin and G. Mathias (2015). "Expanding availability of safe abortion services through private sector accreditation: a case study of the Yukti Yojana program in Bihar, India." Reproductive Health **12**: 1-11.

17. Barber, S. L., S. M. Bertozzi and P. J. Gertler (2007). "Variations in prenatal care quality for the rural poor in Mexico." Health Affairs **26**(3): w310-323.

18. Béhague, D. P., H. Gonçalves and J. Dias da Costa (2002). "Making medicine for the poor: primary health care interpretations in Pelotas, Brazil." Health Policy & Planning **17**(2): 131-143.

19. Boller, C., K. Wyss, M. Deo and M. Tanner (2003). "Quality and comparison of antenatal care in public and private providers in the Unitd Republic of Tanzania." World Health Organization. Bulletin of the World Health Organization **81**(2): 116-122.

20. Chirdan, O. O., L. A. Lar, T. O. Afolaranmi, E. O. Inalegwu, C. S. Igoh and G. U. Adah (2013). "Client satisfaction with maternal health services comparism between public and private hospitals in Jos Nigeria." Jos Journal of Medicine **7**(1): 1-9.

21. d'Orsi, E., O. M. Bruggemann, C. S. Diniz, J. M. Aguiar, C. R. Gusman, J. A. Torres, A. Angulo-Tuesta, D. Rattner and R. M. Domingues (2014). "Social inequalities and women's satisfaction with childbirth care in Brazil: a national hospital-based survey." Cad Saude Publica **30 Suppl 1**: S1-15.

22. Danel, I. and G. Forgia (2005). "Contracting for basic health care in rural Guatemala - Comparison of the performance of three delivery models." Health Systems Innovations in Central America: Lessons and Impact of New Approaches: 49-88.

23. De Savigny, D., C. Mayombana, E. Mwageni, H. Masanja, A. Minhaj, Y. Mkilindi, C. Mbuya, H. Kasale and G. Reid (2004). "Care-seeking patterns for fatal malaria in Tanzania." Malaria Journal **3**: 27.

24. Diamond-Smith, N., M. Sudhinaraset and D. Montagu (2016). "Clinical and perceived quality of care for maternal, neonatal and antenatal care in Kenya and Namibia: the service provision assessment." Reproductive Health **13**: 1-13.

25. Do, M. and S. Agha (2009). Differences in the quality of reproductive health services provided by private midwives in Uganda. MD, Private Sector Partnerships-One project, Abt Associates Inc.**:** 24 p.

26. Duggal, R. and V. Ramachandran (2004). "The Abortion Assessment Project-India: Key Findings and Recommendations." Reproductive Health Matters **12**(24): 122-129.

27. EPHPP. (2010). "Quality assessment tool for quantitative studies. Effective Public Health Practice Project, Hamilton." Retrieved 21 December, 2020, from <https://merst.ca/ephpp/>. .

28. Health Partners International and Montrose (2015). NU Health. Results based financing with non-state providers: insights from a controlled trail in Northern Uganda. Technical Annexes. Kampala (Uganda), NU Health Programme**:** 32 p.

29. Hulton, L. A., Z. Matthews and R. W. Stones (2007). "Applying a framework for assessing the quality of maternal health services in urban India." Social Science & Medicine **64**(10): 2083-2095.

30. Jallow, I. K., Y. J. Chou, T. L. Liu, N. Huang, I. K. Jallow, Y.-J. Chou, T.-L. Liu and N. Huang (2012). "Women's perception of antenatal care services in public and private clinics in the Gambia." International Journal for Quality in Health Care **24**(6): 595-600.

31. Karki, C., M. Ojha and R. T. Rayamajhi (2009). "Baseline survey on functioning of abortion services in government approved CAC centers in three pilot districts of Nepal." Kathmandu Univ Med J (KUMJ) **7**(25): 31-39.

32. Kojima, N., C. C. Bristow, N. Pollock, P. Crouse, H. Theodore, J. Bonhomme, C. F. Gaston, J. G. Devieux, J. W. Pape and J. D. Klausner (2015). "Rapid Training and Implementation of the Pollock Technique, a Safe, Effective Newborn Circumcision Procedure, in a Low-Resource Setting." Glob Pediatr Health **2**: 2333794x15589114.

33. Levin, A., S. Munthali, V. Vodungbo, N. Rukhadze, K. Maitra, T. Ashagari and L. Brenzel (2019). "Scope and magnitude of private sector financing and provision of immunization in Benin, Malawi and Georgia." Vaccine **37**(27): 3568-3575.

34. MacFarlane, K. A., M. L. O'Neil, D. Tekdemir, A. M. Foster and M. L. O'Neil (2017). ""It was as if society didn't want a woman to get an abortion": a qualitative study in Istanbul, Turkey." Contraception **95**(2): 154-160.

35. Mahar, B., R. Kumar, N. Rizvi, H. A. Bahalkani, M. Haq and J. Soomro (2012). "Quantity and quality of information, education and communication during antenatal visit at private and public sector hospitals of Bahawalpur, Pakistan." Journal of Ayub Medical College, Abbottabad : JAMC **24**(3-4): 71-74.

36. Penn-Kekana, L., T. Powell-Jackson, M. Haemmerli, I. L. Lange, G. Sharma, C. Goodman, V. Dutt, K. Singh, V. Shukla, A. Mahapatra and S. Singh (2018). "Process evaluation of a social franchising model to improve maternal health: evidence from a multi-methods study in Uttar Pradesh, India." Implementation Science **13**(124): 1-15.

37. Rahman, M., U. Rob and T. Kibria (2009). Implementation of maternal health financial scheme in rural Bangladesh.

38. Ramachandar, L. and P. J. Pelto (2002). "The role of village health nurses in mediating abortions in rural Tamil Nadu, India." Reprod Health Matters **10**(19): 64-75.

39. Schooley, J., C. Mundt, P. Wagner, J. Fullerton and M. O'Donnell (2009). "Factors influencing health care-seeking behaviours among Mayan women in Guatemala." Midwifery **25**(4): 411-421.

40. Sharma, G., T. Powell-Jackson, K. Haldar, J. Bradley and V. Filippi (2017). "Quality of routine essential care during childbirth: clinical observations of uncomplicated births in Uttar Pradesh, India." World Health Organization. Bulletin of the World Health Organization **95**(6): 419-429.

41. SHOPS Program (2018). Sustainable strategies for accessible, quality health care: Public-private sector engagement in Kenya, Rockville, Maryland, Sustaining Health Outcomes through the Private Sector Plus [SHOPS Plus], Abt Associates Inc., 2018 Aug.**:** 12 p.

42. Sieverding, M., C. Briegleb and D. Montagu (2015). "User experiences with clinical social franchising: qualitative insights from providers and clients in Ghana and Kenya." BMC Health Services Research **15**(1): 49.

43. Solnes Miltenburg, A., Y. Roggeveen, M. v. Elteren, L. Shields, J. Bunders, J. v. Roosmalen and J. Stekelenburg (2013). "A protocol for a systematic review of birth preparedness and complication readiness programs." Systematic Reviews **2**(1): 11.

44. Turan, J. M., A. Bulut, H. Nalbant, N. Ortayli and A. A. Akalin (2006). "The quality of hospital-based antenatal care in Istanbul." Stud Fam Plann **37**(1): 49-60.

45. Vora, K. S., S. L. Saiyed and D. V. Mavalankar (2018). "Quality of Free Delivery Care among Poor Mothers in Gujarat, India: A Community-Based Study." Indian Journal of Community Medicine **43**(3): 224-228.

46. Walsh, D. and S. Downe (2006). "Appraising the quality of qualitative research." Midwifery **22**(2): 108-119.

47. Wendot, S., R. H. Scott, I. Nafula, I. Theuri, E. Ikiugu and K. Footman (2018). "Evaluating the impact of a quality management intervention on post-abortion contraceptive uptake in private sector clinics in western Kenya: a pre- and post-intervention study." Reprod Health **15**(1): 10.

48. Zaidi, S., A. Riaz, F. Rabbani, S. I. Azam, S. N. Imran, N. A. Pradhan and G. N. Khan (2015). "Can contracted out health facilities improve access, equity, and quality of maternal and newborn health services? Evidence from Pakistan." Health Research Policy & Systems **13**(1): 54-54.
