# Supplementary material for: Experiences of private sector quality care amongst mothers, newborns, and children in low- and middle-income countries: a systematic review
Source: BMC Health Serv Res. 2021 Dec 6;21:1311. doi: 10.1186/s12913-021-06905-3 (PMC8647361; doi:10.1186/s12913-021-06905-3)
Supplement: Supplementary file 2 — Additional file 2. [file 12913_2021_6905_MOESM2_ESM.docx]

**Supplementary Annex 2: Intervention characteristics amongst studies reporting specific interventions that went beyond the generic delivery of quality care**

| *Characteristics* | *Number of specific intervention studies included in final inventory (%)* | *Number of specific intervention studies examining experience of care (%)* |
| --- | --- | --- |
| **Intervention quantity** |  |  |
| Single intervention | 42 (72.4%) | 13 (86.7%) |
| Multiple interventions | 16 (27.6%) | 2 (13.3%) |
| **Focus on supply-side or demand-side factors** |  |  |
| Supply-side | 37 (63.8%) | 11 (73.3%) |
| Demand-side | 2 (3.5%) | 1 (6.7%) |
| Both | 18 (31.0%) | 3 (20.0%) |
| Unclear | 1 (1.7%) | -- |
| **Intervention type: on-site support for QI** |  |  |
| No | 27 (46.6%) | 5 (33.3%) |
| Yes | 31 (53.4%) | 10 (66.7%) |
| **Intervention type: data systems** |  |  |
| No | 43 (74.1%) | 13 (86.7%) |
| Yes | 15 (25.9%) | 2 (13.3%) |
| **Intervention type: learning systems** |  |  |
| No | 29 (50.0%) | 10 (66.7%) |
| Yes | 29 (50.0%) | 5 (33.3%) |
| **Intervention type: program management** |  |  |
| No | 29 (50.0%) | 8 (53.3%) |
| Yes | 29 (50.0%) | 7 (46.7%) |
| **Intervention type: advocacy** |  |  |
| No | 31 (53.4%) | 10 (66.7%) |
| Yes | 27 (46.6%) | 5 (33.3%) |
| **Intervention type: policy and strategy development** |  |  |
| No | 46 (79.3%) | 13 (86.7%) |
| Yes | 12 (20.7%) | 2 (13.3%) |
| **Intervention recipient: women during pregnancy, childbirth, and/or postpartum** |  |  |
| Indirect recipient | 10 (17.2%) | 3 (20.0%) |
| Direct recipient | 16 (27.6%) | 5 (33.3%) |
| Not a recipient | 32 (55.2%) | 7 (46.7%) |
| **Intervention recipient: women defined more generally** |  |  |
| Indirect recipient | 9 (15.5%) | 3 (20.0%) |
| Direct recipient | 4 (6.9%) | 1 (6.7%) |
| Not a recipient | 45 (77.6%) | 11 (73.3%) |
| **Intervention recipient: infants** |  |  |
| Indirect recipient | 11 (19.0%) | 2 (13.3%) |
| Direct recipient | 7 (12.0%) | 1 (6.7%) |
| Not a recipient | 40 (69.0%) | 12 (80.0%) |
| **Intervention recipient: children** |  |  |
| Indirect recipient | 10 (17.2%) | 1 (6.7%) |
| Direct recipient | 11 (19.0%) | 1 (6.7%) |
| Not a recipient | 37 (63.8%) | 13 (86.7%) |
| **Intervention recipient: health care providers** |  |  |
| Indirect recipient | 2 (3.4%) | 0 (0%) |
| Direct recipient | 49 (84.5%) | 14 (93.3%) |
| Not a recipient | 7 (12.1%) | 1 (6.7%) |
| **Type of intervention evaluation** |  |  |
| Impact | 26 (45.6%) | 8 (53.3%) |
| Process | 12 (21.1%) | 2 (13.3%) |
| Impact and process | 15 (26.3%) | 4 (26.7%) |
| Multiple (e.g., impact, process, economic) | 4 (7.0%) | 1 (6.7%) |
